# Using routinely collected clinical data for circadian medicine: A review of opportunities and challenges

**DOI:** 10.1371/journal.pdig.0000511

**Published:** 2024-05-23

**Authors:** Laura Kervezee, Hassan S. Dashti, Luísa K. Pilz, Carsten Skarke, Marc D. Ruben

**Affiliations:** 1 Group of Circadian Medicine, Department of Cell and Chemical Biology, Leiden University Medical Center, Leiden, the Netherlands; 2 Department of Anesthesia, Critical Care and Pain Medicine, Massachusetts General Hospital and Harvard Medical School, Boston, Massachusetts, United States of America; 3 Department of Anesthesiology and Intensive Care Medicine CCM / CVK, Charité–Universitätsmedizin Berlin, corporate member of Freie Universität Berlin and Humboldt Universität zu Berlin, Berlin, Germany; 4 ECRC Experimental and Clinical Research Center, Charité–Universitätsmedizin Berlin, corporate member of Freie Universität Berlin and Humboldt Universität zu Berlin, Berlin, Germany; 5 Institute for Translational Medicine and Therapeutics (ITMAT), University of Pennsylvania Perelman School of Medicine, Philadelphia, Pennsylvania, United States of America; 6 Chronobiology and Sleep Institute (CSI), University of Pennsylvania Perelman School of Medicine, Philadelphia, Pennsylvania, United States of America; 7 Department of Medicine, University of Pennsylvania Perelman School of Medicine, Philadelphia, Pennsylvania, United States of America; 8 Divisions of Pulmonary and Sleep Medicine and Biomedical Informatics, Cincinnati Children’s Hospital Medical Center, Cincinnati, Ohio, United States of America; University of Michigan, UNITED STATES

## Abstract

A wealth of data is available from electronic health records (EHR) that are collected as part of routine clinical care in hospitals worldwide. These rich, longitudinal data offer an attractive object of study for the field of circadian medicine, which aims to translate knowledge of circadian rhythms to improve patient health. This narrative review aims to discuss opportunities for EHR in studies of circadian medicine, highlight the methodological challenges, and provide recommendations for using these data to advance the field. In the existing literature, we find that data collected in real-world clinical settings have the potential to shed light on key questions in circadian medicine, including how 24-hour rhythms in clinical features are associated with—or even predictive of—health outcomes, whether the effect of medication or other clinical activities depend on time of day, and how circadian rhythms in physiology may influence clinical reference ranges or sampling protocols. However, optimal use of EHR to advance circadian medicine requires careful consideration of the limitations and sources of bias that are inherent to these data sources. In particular, time of day influences almost every interaction between a patient and the healthcare system, creating operational 24-hour patterns in the data that have little or nothing to do with biology. Addressing these challenges could help to expand the evidence base for the use of EHR in the field of circadian medicine.

## Introduction

Thousands of data points may be generated and digitally stored each day a patient is in the hospital [1]. These electronic health records (EHR) often include vital signs, imaging and laboratory results, diagnoses, medications, medical history, demographic information, and clinical notes. Although EHR, by definition, are built for patient care [2], the data are increasingly used to address a wide range of biomedical research questions.

One area of research that may benefit from routinely collected clinical data is the burgeoning field of circadian medicine, which aims to translate findings from circadian biology to clinical practice. Owing to their high resolution and longitudinal nature, EHR data have been referred to as a “treasure trove of information” that can support all facets of research in circadian medicine [3]. However, repurposing real-world clinical data for biomedical research requires a great deal of consideration. In addition to the general challenges facing all EHR-based research [4–6], efforts to understand the role of time of day present unique challenges, which have received only limited attention.

In this narrative review, our goal is to (i) provide an overview of the potential for EHR to advance circadian medicine; (ii) to highlight the challenges pertaining to responsible use of these data; and (iii) offer recommendations for researchers who are planning to use EHR data in the field of circadian medicine.

## Literature search methodology

This narrative review was prepared by identifying peer-reviewed articles in electronic databases (PubMed and Google Scholar) using search terms including “circadian” and “electronic health records” or “routinely-collected clinical data” and variants of those terms. Inclusion criteria were research articles that describe 24-hour patterns in physiological readouts collected using routinely collected clinical data, focusing on EHR data from inpatient hospital stays since these patients are monitored around the clock. However, many of the points we discuss are applicable to EHR data from outpatient clinics, disease registries, and other sources. In addition, to provide an overview of the limitations and challenges related to the use of electronic health records, we searched PubMed and Google Scholar for peer-reviewed articles on this topic. In principle, all literature available on these topics were considered without an explicit date range, although priority was given to recent literature due to constraints in word count and number of references set by the journal.

## Daily rhythms in physiology

Human physiology and behavior change profoundly between day and night. The most obvious example is the sleep–wake cycle, but also a broad range of other behaviors (e.g., food intake, mood) and physiology including cardiac (e.g., blood pressure, heart rate, thrombus formation), metabolic (e.g., glucose and lipid metabolism, insulin secretion, metabolic rate), immune and inflammatory (e.g., cytokine secretion, circulating leukocytes), gastrointestinal (e.g., digestion, absorption, and electrolyte balance), thermoregulatory, and hormonal functions vary with predictable patterns over 24 hours [7–12]. Consequently, many clinical tests or measures may depend on the time of day of data collection.

These daily fluctuations in physiology are in part driven by the circadian clock [13], an internal timing system built from 24-hour molecular oscillators that exist in virtually every cell in the body [14]. These oscillators drive circa 24-hour rhythms in gene transcription and translation that synchronize with salient 24-hour rhythms in the environment, such as light–dark or feeding-fasting cycles. In this way, the circadian system anticipates predictable changes in the environment and helps to enact the right physiology at the right time, while also keeping an internal temporal order [15]. For example, blood pressure follows a 24-hour rhythm characterized by a morning rise, afternoon peak, and overnight dip, with a typical daily variation from 10% to 20% over the course of a day [16]. This variation is thought to reflect the differing demands for blood flow during the day (physical and mental exertion) versus the night (recovery, repair, efficiency) [17].

Circadian rhythms can be uncovered in highly controlled laboratory conditions using constant routine or forced desynchrony protocols [18,19]. For example, core body temperature exhibits endogenous circadian rhythmicity that continues to oscillate with a period of approximately 24 hours, even in the absence of external timing cues [20,21]. Under entrained conditions, i.e., in the presence of cues from a 24-hour light–dark cycle, circadian-regulated physiology will cycle with a period of exactly 24 hours. However, the endogenous circadian cycle is not the only contributor to day–night variation in physiology. Changes in activity, posture, meals, sleep, room temperature, light, and potentially many other factors can contribute to daily patterns in physiology [19]. For example, any periodic fluctuation in core body temperature observed in daily life may be caused by diet-induced thermogenesis or physical activity, leading to an increase in core body temperature during the active phase when food intake occurs and a decrease during the resting phase. In the context of distal skin temperature rhythmicity, the peak-to-trough difference of the endogenous circadian rhythm, measured in highly controlled conditions, is about 3.5°C, while this is 6°C in freely living conditions, likely conditioned by behavioral and environmental components [22]. In clinical settings, medical activities such as drug administration, mechanical ventilation, nutritional support, or physical therapy as well as excess sound and artificial light may impose daily rhythms on physiology or completely mask or abolish them. Although the relevance of the interaction between circadian rhythms and behavioral and environmental factors remains to be determined, conceptually, EHR data offer an opportunity to study this at scale and close to the clinical reality. Patient-derived data are inherently noisier than data collected from well-controlled experiments but may contain time-specific signals that are modulated by the circadian clock-behavioral-environmental interaction. The question is: Can novel variables be extracted from this parameter space and would these be indicative of the patient’s clinical status? Here, the massive amounts of data stored in EHR offer opportunities to evaluate this in the context of clinical care.

## Electronic health records for research

EHR databases store all of the electronic data collected in clinical care. Although EHR are primarily used for patient care (e.g., to inform clinical decision-making) and administrative purposes (e.g., billing and insurance), these data are increasingly being applied to biomedical research questions. The advantages of EHR seem clear: (1) data are readily available, reducing costs and burden; (2) data are systematically captured from all patients within the healthcare system, reducing inclusion bias and potentially improving generalizability; (3) databases are large and rapidly grow with time, enabling the ascertainment of case–control patient cohorts, assessment of interactions, and subgroup analyses; (4) data are collected over prolonged periods of time, enabling longitudinal analyses; (5) data are collected in real-world conditions, improving ecological validity [23]; and (6) data are multimodal, enabling analyses along many health measures—medical and nonmedical. However, such advantages should be viewed with caution as EHR are not primarily intended for research.

Many factors can compromise the validity and generalizability of EHR-based research, as has been reviewed elsewhere [5,24]. As patients interact with a healthcare system, EHR documents what happens and when it happens. However, EHR does not explicitly document the why. Hospital admissions, tests, treatment, and discharge are the result of complex decisions made by the patient, clinical staff, and healthcare system. This underlies a key challenge in EHR analysis: The decision-making (data-generating) process is often unknown. This may result in sample selection bias, whereby the presence of data is related to its outcome, as a measurement is only made with clinical justification. Similarly, sampling frequency is inevitably correlated with disease severity; monitoring is more frequent for sicker patients. Furthermore, information within the EHR may be imprecisely defined or related to billing rather than clinical purpose resulting in patient misclassification. For example, a diagnostic code for sleep apnea is necessary for the reimbursement of a sleep apnea exam but is not always indicative of sleep apnea onset [25]. These forms of bias and confounding should be considered when undertaking EHR-based research; a multidisciplinary team with clinical, epidemiological, and statistical expertise is indispensable.

Despite these challenges, EHR-based research is increasing dramatically, in part owing to the release of public databases with deidentified data [26] and technological advances that allow researchers to use EHR from their own or collaborating institutions. Notable examples of open-access databases in the intensive care domain are the MIMIC-IV database [27], the eICU [28], the HiRID database [29], and the AmsterdamUMCdb [30]. Before embarking on a research project that involves setting up a new database derived from EHR, it is important to be aware that this process is time-consuming, labor-intensive, and will undoubtedly raise nontrivial and unexpected issues, as recently described in an honest account of the challenges encountered while preparing EHR data for secondary use [31]. Extraction and curation of EHR data requires a specific set of skills, including expertise with (i) usability; (ii) data quality and validation; (iii) standards for data structuring; (iv) governance (including ethical, security, and privacy aspects related to the responsible use of clinical data); and (v) data integration [32,33]. Therefore, the ease with which one can tap into EHR data within an institute depends on the infrastructure and (programming) support that is available as well as the local legal and privacy regulations [34].

## Opportunities for the use of EHR in circadian medicine

**Fig 1:**
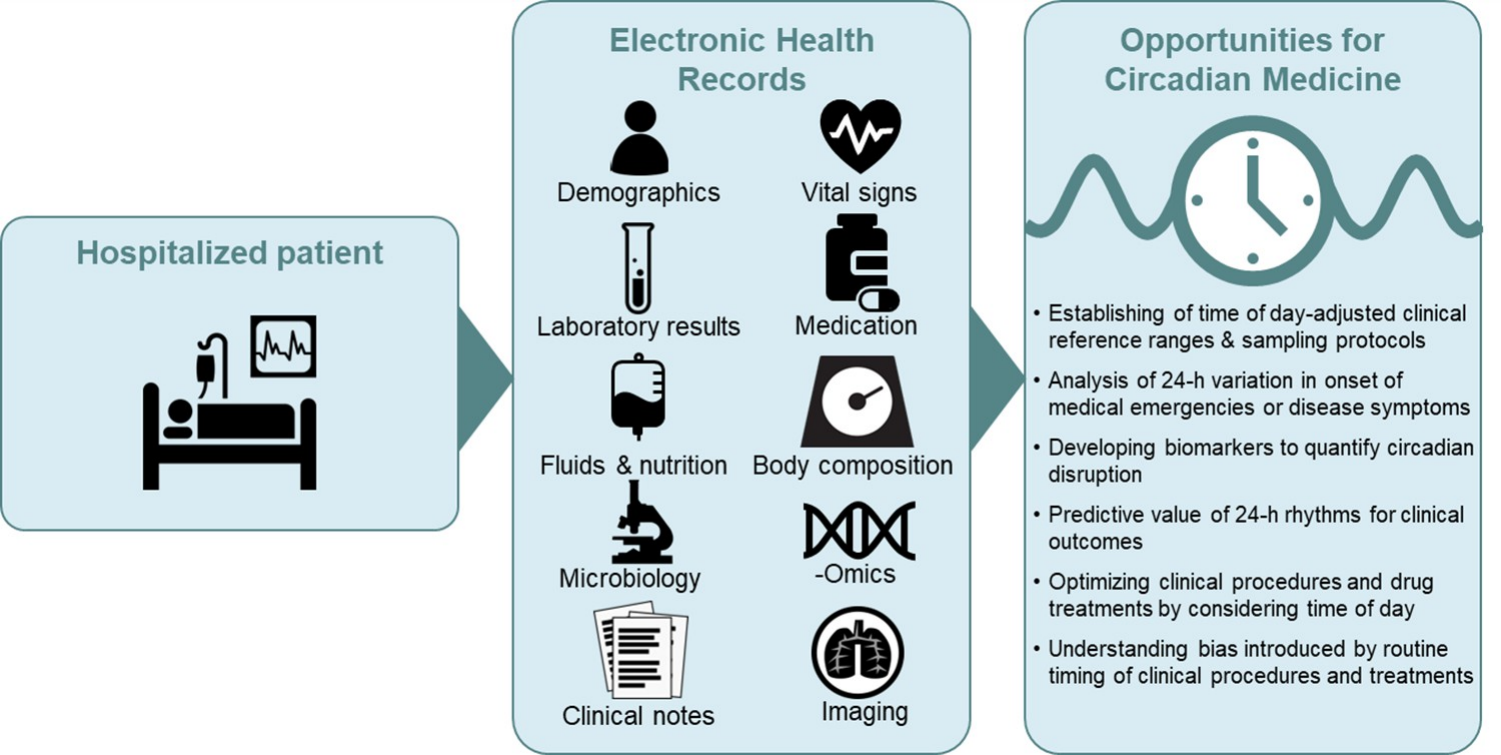
Overview of opportunities for the use of EHR for circadian medicine.

EHR can be used to address key questions in circadian medicine (**Fig 1**). Variables that are routinely monitored in patients show robust 24-hour rhythms in healthy individuals, such as vital signs (e.g., heart rate, blood pressure, and core body temperature) as well as biochemical, metabolic, and hematological factors [35–40]. Using EHR, researchers can evaluate these features in any disease context, providing real-world evidence for the hypothesis that circadian rhythms are a marker of healthy physiological functioning and that altered rhythms indicate poor health [41]. For example, an abnormal blood pressure rhythm, in particular the loss of overnight dipping, has been associated with an increased risk of death and cardiovascular events [42]. An EHR-based study of approximately 60,000 primary care patients found that nighttime systolic blood pressure is more informative about the risk of death than clinic or daytime ambulatory blood pressure, underscoring the value of large 24-hour datasets [43].

The effects of disease onset and hospitalization on 24-hour physiology have historically been evaluated in small, well-defined groups of patients [44–47]. Recent studies are tackling this question using large-scale, open-access EHR databases. For example, a study encompassing nearly 200,000 ICU patients spanning multiple hospitals in the United States of America and the United Kingdom detected population-level 24-hour rhythms in systolic blood pressure, heart rate, respiratory rate, and body temperature in the 24 hours prior to patient discharge. These rhythms were comparable to rhythms in healthy individuals, albeit with reduced peak-to-trough excursions [48]. This raises many questions, potentially also evaluable with EHR. For example, do these peak-to-trough excursions correlate with patient health status, and if so, can they be used as part of the decision-making process in patient discharge? EHR studies suggest that reduced day–night differences in vital signs are associated with increased mortality in hospitalized patients [49–53], but not all studies agree [54]. Future research is needed to understand if and how 24-hour physiology tracks health and predicts outcomes.

EHR-based studies of biomarker variation may also help to refine reference ranges [55]. Analysis of approximately 500,000 blood draws found that the reference interval for thyroid hormone (the range covering the central 95% of results) differs based on age, sex, ethnicity, and time of day [56]. Others have suggested that the time of day of sampling may be an important factor when assessing glucose control in ICU patients [57,58] or to help minimize under- or overdiagnosis in the context of adrenal gland disorders [59]. To speculate, the information gained from any laboratory test about the clinical status of a patient may improve with an understanding of the expected 24-hour dynamics, analogous to the case for blood pressure discussed above. EHR offers huge amounts of data to evaluate this, although analysis of time of day for blood draws is not without its challenges, as discussed in a subsequent section.

A key goal of circadian medicine is to optimize the timing of medication and clinical procedures [3]. Understanding how time of day modifies treatment efficacy and safety may lead to cost-effective and noninvasive timing-based strategies [60,61]. Although randomized controlled trials (RCTs) will remain the gold standard in this regard, current RCTs have some limitations. A survey of RCTs comparing the effects of medication taken at different times of day [62] pointed out that many studies (i) did not account for individual variation in circadian phase (e.g., 8 AM is not morning-time for a shift worker); (ii) often compare only 2 dosing times, morning versus evening; and (iii) were small and single-sited, leading to concerns about their replicability. Exploratory studies using EHR offer a practical solution to some of these shortcomings and may be used to guide confirmatory RCTs. For example, using EHR from a pediatric hospital, it was found that the clinical response to hydralazine, an acute antihypertensive, is greatest at night [63].

Further, by analyzing breakthrough Coronavirus disease 2019 (COVID-19) infection in 1.5 million patients as a function of time of vaccination, late morning to early afternoon was found to offer better protection than evening vaccination [64]. The authors of this study were able to construct a continuous efficacy curve over a 12-hour period using EHR data. It is difficult to imagine an RCT achieving similar temporal resolution at this scale. These examples highlight the potential of everyday healthcare data to deliver insights into the impact of time on treatment and many other health measures.

In addition to considering time of day as a source of biological variation, the interaction between time of day and clinical routines, or healthcare processes, is another aspect of circadian medicine. Although clinical care takes place around the clock in hospitals, specific procedures or treatments are provided at specific times of day and not at other times. The timing of medication orders and administration are influenced by hospital rounding and may not be driven by the time of greatest clinical need [65,66]. Similarly, sampling for laboratory tests typically occurs in the early morning hours [67,68] and other care activities are—not surprisingly—unevenly distributed around the clock [69]. Given that all this information is typically documented in EHR, there is an opportunity to study how healthcare processes interact with biological variation.

We expect EHR-based circadian medicine research to grow as technological integration evolves. Wearable devices, for example, extend close monitoring of physiological and behavioral variables beyond the clinic into patient communities under real-world conditions. First evidence for this seamless integration of EHR with wearable devices are emergent [70,71], including wrist accelerometers [72] and sleep-monitoring technologies [73]. Wearable technology for longitudinal recording of physiological or behavioral variables is extensively adopted in the field of chronobiology to characterize 24-hour patterns in variables of interest. Some examples include activity trackers [74,75], continuous glucose monitoring [76], wrist temperature [22], and heart rate [76,77]. The relevance of using wearables for circadian monitoring in patient populations is exemplified by studies showing the predictive value of circadian metrics and clinical outcomes in patient populations, such as circadian features of heart rate as a prognostic marker for postoperative recovery scores [78] and rest–activity cycles as a predictor for patient survival [79] in oncology patients.

Furthermore, integration of molecular information with EHR will also pave the way for precision medicine [80]. For example, variation in circadian genes or genetic profiles related to circadian rhythms could inform treatment and risk prediction. In addition, further validation of blood-based circadian phenotyping using a single blood sample may allow for accurate determination of a patient’s internal circadian time based on transcriptional biomarkers [80,82]. How best to incorporate those data with EHR and how to make this clinically actionable remains to be determined.

## Challenges for the use of EHR for circadian medicine

**Fig 2:**
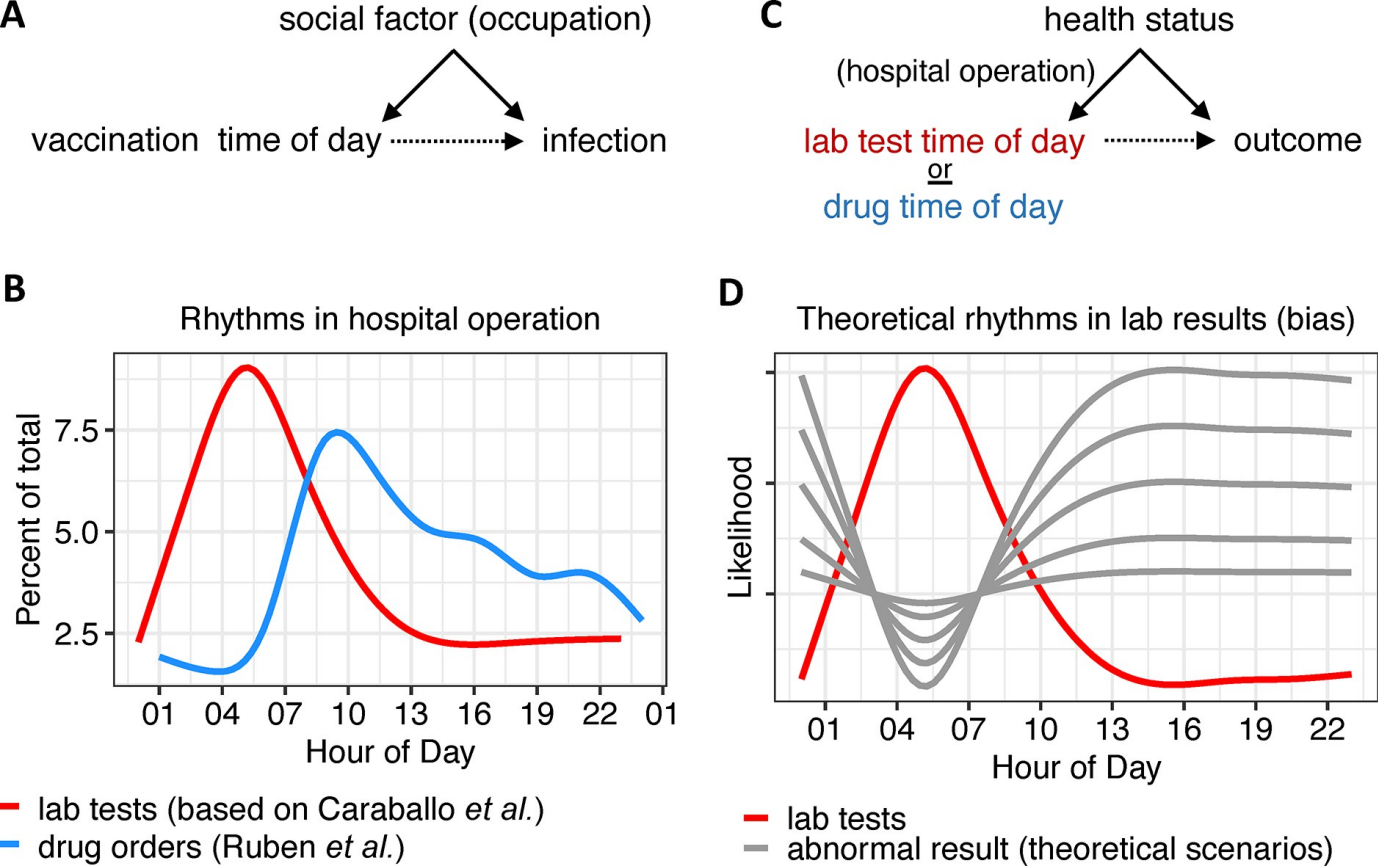
Considering causes in EHR studies of biological rhythms. **(A)** An individual’s occupation may independently “cause” both the time of vaccination (scheduling/availability) and the risk of COVID-19 infection (exposure). A way to think about cause in these diagrams is that the variable being pointed to “listens to” the variable pointing to it. **(B)** Twenty-four-hour rhythms in hospital operation based on data from Ruben and colleagues [65] and Caraballo and colleagues [68]. **(C)** Proposed causal model for the effect of time of day of lab test (or drug administration) on outcome. This model assumes that health status is a confounder because it independently influences both the time of lab test (or drug administration) and the outcome of interest. **(D)** Given the causal model for lab results in panel C, we propose that the probability of an abnormal result at a particular time is inversely related to the probability of testing at that time. Theoretical curves (gray color) reflect different scaling, i.e., proposed relationships between the probability of testing and probability of obtaining an abnormal result. Ground truth is unknown.

EHR studies use data that were not collected to address predefined research questions and thus require a great deal of care in analysis and interpretation. Besides pitfalls that pertain to research with EHR in general, the use of these databases for circadian medicine purposes introduces multiple challenges (**Table 1**). The COVID-19 vaccine timing study [64] offers a good example. In that study, an individual’s occupation may have independently influenced both the time of vaccination and the risk of COVID-19 infection (**Fig 2A**). To account for this, the authors carefully estimated a baseline infection rate for each comparison group by calculating COVID-19 positivity in the 14 days after the first vaccine dose, before any protection from the vaccine is expected. Although confounding is impossible to rule out, considering all of the potential causal relationships in the data and measuring them whenever possible increases the power and interpretability of EHR analyses.

**Table 1:** Challenges and recommendations on applying EHR to studies of circadian medicine.

Challenges	Recommendations	Guiding questions
**Sample selection bias** EHR are often collected from specific healthcare systems or patient populations. As such, it may not represent the entire population.	Always remember that data collection was not guided by research questions and is greatly influenced by the functioning of the healthcare institution.	• Have we properly discussed external validity and generalizability?
**Heterogeneity in data collection** Procedures and type of data collected (e.g., which patients, sampling frequency, different technologies) heavily depend on clinical routines and administrative purposes.	Consider potential biases by drawing out causal diagrams, and engaging domain and statistical expertise before, during, and after analysis.	• Could daily patterns of hospital operation have imprinted daily patterns of clinical values? • Are there hidden variables (e.g., disease severity) that may have influenced both the time of day of measurement and the outcome of interest (e.g., white blood cell count)? • Which of all the potentially causal variables can we account for (and not account for) in our analysis?
**Variability in data storage/management** Data management practices may vary significantly across institutions.	Confirm that event date-time stamps are reliable.	• Were date-times shifted to protect privacy? • Could there be a significant date-time lag between the event and its electronic entry?
	Consider whether the clinical features important to your study may be imprecisely defined.	• Could the diagnosis code that we used to define a cohort have been assigned primarily for billing purposes? How might this affect my results?
**Data structure/types** Multilevel data: Data may involve a single measure from each of N patients or multiple measures from each of N patients.	Consider the independence of observations in your dataset.	• Have we accounted for hierarchical structure in our dataset? For example, repeated hospital admissions for the same patient cannot be treated as independent observations. Similarly, repeated measures on a patient during an admission cannot be treated as independent observations.
Time of day is a circular variable: It represents a cyclical phenomenon.	Avoid discretizing circular variables.	• Can we treat time of day as a circular variable in order to avoid binning observations into wide intervals (e.g., day vs. night)?
**Reproducibility-related challenges** EHR data has sensitive patient information, and strict privacy regulations have to be followed when assessing, using and sharing these data for research purposes. Data are often stored in different formats and systems, which make integration difficult. Preprocessing of data can be undertaken in different ways.	Follow best-practice statements developed specifically for EHR-based studies.	• Was CODE-EHR or a similar framework used when planning and conducting the study?
	Determine how to share data, code, and software needed for someone else to recreate findings and figures.	• How do we legally and responsibly share the data and code? • How do we future-proof this access, for example, how do we prevent broken web links? • How do we meet the requirements for journals’ data/code availability statements?
	When drafting a manuscript, follow appropriate reporting guidelines.	• Have we checked RECORD (observational) or CONSORT-ROUTINE (intervention studies)? • Did we openly discuss the biases we are aware of (there are always plenty of them) in our manuscript?

It is easy to underestimate the degree to which time of day influences the interaction between a patient and the healthcare system. Two recent studies highlight how treatment [65] and diagnosis [68] follow well-established 24-hour patterns of care in the hospital (**Fig 2B**). For example, blood and urine specimens are routinely collected in the early morning hours so that the test results are available for clinical team rounding that typically occurs 3 to 4 hours later. Of course, not all specimens are collected in this routine window. In particular, a patient with acutely concerning signs or symptoms (and, therefore, a higher likelihood of abnormal test values compared to more stable patients) may receive off-peak testing. To this point, the time of day that a lab test is ordered predicts mortality, in some cases even more powerfully than the test result itself [67]. In other words, the decision to acquire data is influenced by clinicians’ perception of clinical need in a background of operational routine (**Fig 2C and 2D**). As a consequence, it may be easy to mistake patterns in hospital operations for rhythms in biology (**Fig 2D**). For example, 24-hour variation in glucose levels in EHR data from critically ill patients was initially attributed to circadian variation in glucose control [57] but was later shown to be—at least partially—caused by more intensive sampling in sicker patients [58]. When using time-weighted averages, the time of day effect was greatly attenuated [58].

Considering the potential influences on an outcome, drawing causal diagrams [83], and engaging domain expertise can help to clarify the clinical process and determine the best statistical approach [84]. Propensity score matching may be used to ensure patient subgroups are comparable for analyses [85]. Still, propensity matching requires (1) awareness of potential causal factors and (2) a way to measure them. This can be hard. For example, we may be aware that health status causes the collection of lab values and, therefore, try to “match” health status between comparison groups. But how do we measure health status? Can we disentangle these factors, given that time of day of testing predicts health status, in some cases even better than the test results themselves [67]? This is an interesting form of confounding that makes it difficult to ever fully control for health status when trying to study biological rhythms in lab values. A similar issue may present in studies on medication timing (**Fig 2B**) where treatment received “off peak” may describe different types of patient.

Inpatients may receive automated 24-hour continuous monitoring of clinical parameters, including blood pressure, heart rate, body temperature, respiratory rate, and oxygen saturation. Automation mitigates some but not all concerns of the bias discussed above. Consider that not all inpatients receive 24-hour monitoring. It is a decision, influenced by operational routine and perceived clinical need. The patient population that is being closely monitored may not be representative of all individuals with the condition of interest. In addition, the use of routine dosing times of drugs may impose 24-hour rhythms in monitored parameters; for example, the administration of vasodilators or vasopressors during a specific time window may produce a 24-hour pattern in blood pressure. It is possible to exclude data points collected within the time frame that drug administration occurs to limit this effect [48]; however this may exclude a substantial amount of data, especially in settings like the ICU where medication use is high.

In the vaccination study above, and other EHR-based assessments of timing effects [48,50], data from many individuals are compressed onto a single hypothetical 24-hour cycle and analyzed. This may involve a single measure from each of N patients or multiple measures from each of N patients. Either strategy helps to “cover the clock” when individual sampling is sparse but can create imbalances in statistical power across the 24-hour cycle. Mixed-effects models or other statistical methods that take into account repeated sampling may be useful to account for interindividual variability in baseline or responses in case patients contribute multiple measures [86]. In addition, it should be noted that time of day is circular and, although it may be tempting to discretize observations into “morning” or “evening” (or something similar), time is best handled as a circular predictor to avoid information loss. The development of statistical methods to describe periodic processes is an active area of research [75,87,88].

As a final point, precise definition of the variables under study is crucial. In circadian medicine, it is important to verify that time of day is correctly specified. This may sound trivial but open-access EHR databases may shift dates and times to protect patient anonymity. For example, while time of day information is preserved in the MIMIC-IV, dates have been shifted by a random number of days, precluding seasonal or day of week analyses [27]. In some databases, time may be parameterized relative to when the patient was first admitted, making it impossible to study time of day effects [30].

## Conclusions

The promise of circadian medicine is clear: There are numerous opportunities to improve diagnostics, treatments, and patient care by incorporating circadian principles. A goal of future research is to expand the evidence base for these approaches in real-world clinical settings. With this goal in mind, we offer a few recommendations for the use of EHR in circadian medicine (see **Table 1**).

First, we recommend evaluating the suitability of variables for circadian research based on data collection methods rather than data type. EHR-based circadian research has mostly focused on vital signs like heart rate, blood pressure, and body temperature. However, as discussed throughout this review, the EHR holds data on numerous other variables that could offer valuable insights into circadian effects on health and disease. These variables include information about patients’ diet, exercise, posture, sleep, light exposure, urinary output, nurse call button usage, pain levels, and much more. The collection process is not always transparent but almost always impacts data quality, resolution, and potential bias, as exemplified by the distinction between automated and nonautomated data collection. The process of clinical data collection is evolving, with technological advances allowing continuous recording of physiology that is currently measured only at limited time points [76,79]. These advances offer promising opportunities for circadian medicine research.

Moreover, it is important to know the intricacies of a database before analyzing its content. Random shuffling of times and dates for deidentification can go unnoticed, jeopardizing studies. Second, responsible sharing of deidentified raw patient-level data according to FAIR (Findable, Accessible, Interoperable, Reusable) principles [90] enhances transparency, accelerates the development of data-driven methods, and allows benchmarking across multiple cohorts, as is done in the field of sleep research [91]. Institutional or national regulations may preclude data sharing, especially in the case of sensitive medical data, although informative examples of data sharing strategies that comply with national privacy regulations exist [30].

To ensure reproducibility, it is considered good practice to share and maintain any code or applications used to process and analyze the data [92]. When embarking on a study involving EHR, we encourage authors to follow best-practice statements that have been specifically developed for this purpose, such as the CODE-EHR framework [93]. Likewise, when drafting a manuscript, reporting guidelines intended for studies using routinely collected clinical data should be followed, such as the RECORD guidelines for observational studies [2] or the CONSORT-ROUTINE guidelines for intervention studies [94]. This will enhance transparency and reproducibility of the study and facilitate systematic reviews and meta-analyses.

In general, we expect that the opportunities, challenges, and recommendations discussed in this review will help to future-proof the research in the field of circadian medicine. EHR may indeed be a treasure trove for the field, but—like real treasure troves—only if they are handled with care.
